# A guaranteed income intervention to improve the health and financial well-being of low-income black emerging adults: study protocol for the Black Economic Equity Movement randomized controlled crossover trial

**DOI:** 10.3389/fpubh.2023.1271194

**Published:** 2023-11-03

**Authors:** Sheri A. Lippman, Margaret K. Libby, Michelle K. Nakphong, Abigail Arons, Monica Balanoff, Adrienne Rain Mocello, Emily A. Arnold, Starley B. Shade, Fahad Qurashi, Alexandria Downing, Alexis Moore, William H. Dow, Marguerita A. Lightfoot

**Affiliations:** ^1^Division of Prevention Science, Department of Medicine, University of California, San Francisco, San Francisco, CA, United States; ^2^MyPath, San Francisco, CA, United States; ^3^Department of Epidemiology and Biostatistics, Institute for Global Health Sciences, University of California, San Francisco, San Francisco, CA, United States; ^4^Department of Health Policy and Management, University of California, Berkeley, Berkeley, CA, United States; ^5^Oregon Health & Science University – Portland State University School of Public Health, Portland, OR, United States

**Keywords:** guaranteed income, Black young adult, economic empowerment, cross-over trial, cash transfer, socioeconomic disparities in health, systemic racism

## Abstract

**Background:**

Economic inequity systematically affects Black emerging adults (BEA), aged 18–24, and their healthy trajectory into adulthood. Guaranteed income (GI)–temporary, unconditional cash payments–is gaining traction as a policy solution to address the inequitable distribution of resources sewn by decades of structural racism and disinvestment. GI provides recipients with security, time, and support to enable their transition into adulthood and shows promise for improving mental and physical health outcomes. To date, few GI pilots have targeted emerging adults. The BEEM trial seeks to determine whether providing GI to BEA improves financial wellbeing, mental and physical health as a means to address health disparities.

**Methods/design:**

Using a randomized controlled crossover trial design, 300 low-income BEA from San Francisco and Oakland, California, are randomized to receive a $500/month GI either during the first 12-months of follow-up (Phase I) or during the second 12-months of a total of 24-months follow-up (Phase II). All participants are offered enrollment in optional peer discussion groups and financial mentoring to bolster financial capability. Primary intention-to-treat analyzes will evaluate the impact of GI at 12 months among Phase I GI recipients compared to waitlist arm participants using Generalized Estimating Equations (GEE). Primary outcomes include: (a) financial well-being (investing in education/training); (b) mental health status (depressive symptoms); and (c) unmet need for mental health and sexual and reproductive health services. Secondary analyzes will examine effects of optional financial capability components using GEE with causal inference methods to adjust for differences across sub-strata. We will also explore the degree to which GI impacts dissipate after payments end. Study outcomes will be collected via surveys every 3 months throughout the study. A nested longitudinal qualitative cohort of 36 participants will further clarify how GI impacts these outcomes. We also discuss how anti-racism praxis guided the intervention design, evaluation design, and implementation.

**Discussion:**

Findings will provide the first experimental evidence of whether targeted GI paired with complementary financial programming improves the financial well-being, mental health, and unmet health service needs of urban BEA. Results will contribute timely evidence for utilizing GI as a policy tool to reduce health disparities.

**Clinical trial registration:**

https://clinicaltrials.gov, identifier NCT05609188.

## Introduction

The significant physical, emotional, and developmental changes that occur during the developmental period of emerging adulthood (ages 18–24) put young people at risk for increased mood disorders, increased risk-taking behaviors, and decreased health-seeking behaviors (1–6). Poverty, driven by deep systemic social and structural inequities, exacerbates these negative outcomes and upends the safe and healthy transition to adulthood (7, 8). The impacts of poverty are acute for Black adolescents and young adults, under the age of 25 years. Compared to White adolescents and young adults, Black adolescents and young adults in the U.S. experience higher levels of poverty, illness, and discrimination (9–11). These exposures to harm, coupled with the lack of supportive services to address and mitigate poverty and structural inequities, result in disproportionately adverse mental and physical health outcomes. Black adolescents and young adults report high mental health service needs (12, 13), but receive services at a lower rate than other racial groups of the same age (14–16)–over half (58%) of Black adolescents and young adults with a serious mental illness report not receiving treatment in the past year (17). In terms of physical health, Black adolescents and young adults account for nearly 60% of new HIV infections within their age group in the U.S. (18), despite comprising 14% of the total adolescent and young adult population. Black adolescents and young adults experience gonorrhea at eight times the rate of their White peers and chlamydia at four times the rate of White peers (19, 20). Disrupting the social determinant of poverty that systematically affects young people during the critical developmental period of emerging adulthood can have a transformative impact on their mental and physical health, and their trajectory into adulthood.

Envisioning a more equitable and healthy future for Black emerging adults (BEA) (ages 18–24) requires bold, multilevel interventions that address structural-level factors, including poverty and racism, and their fundamental impacts on health (21, 22). Structural racism and the resulting socioeconomic inequality has limited the opportunities for Black Americans for generations, through a number of institutional and policy-level mechanisms (i.e., Jim Crow laws and ongoing discrimination in housing, hiring and lending) that reduce access to wealth and health (23–25). Over the past 30 years, the average wealth of White families has grown at three times the rate of growth for Black families (26). Further, economic programs in the U.S. historically have tied income assistance to work (e.g., welfare to work, unemployment), which restricts who can access assistance and how it is used and require lengthy applications (27), all of which obstructs a path out of poverty.

Upending poverty and economic inequity requires providing BEA the security, time, and supports needed to enable their transition into financial independence, including support to invest in long-term financial well-being, such as educational and occupational training. One approach to transforming structural inequities is providing a guaranteed income (GI), without contingencies or burdensome requirements. GI, rooted in racial justice movements (28, 29), has been proposed as a strategy to counteract the effects of structural racism and years of disinvestment in Black communities (30). Pilot GI programs tested with low-income adults have shown impressive gains in economic security that have impacted markers of health and well-being. For example, the Stockton Economic Empowerment Demonstration (SEED) project, a GI program based in Stockton, California, that gave selected residents $500 per month, increased recipients’ full-time employment and decreased anxiety and depression, after 1 year (31). Past GI experiments have found that adolescents in households that received GI were more likely to continue schooling than non-recipient households (32–34). A recent systematic review of guaranteed income studies in high-income countries also found consistent and clear improvements in mental health coupled with reductions in stress and hope for the future (35). Early evidence from these programs have resulted in support for GI projects in cities across the United States (36).

Within the nascent GI movement in the US, GI programs are quickly expanding among some subpopulations of young adults (e.g., young adults experiencing homelessness) (37, 38), although they have rarely been rigorously evaluated during the critical transitional period of emerging adulthood. Programs that did target adolescents and young adults have largely been conditional (requiring engagement in a specific behavior) (39, 40), directed payments to the family rather than adolescents and young adults themselves (40, 41), or were of limited scientific scope and size (42, 43). Providing GI to BEA could transform their opportunities as they develop their educational, employment, and financial trajectories and before financial challenges lead to deepening debts or abandoning educational pathways. Further, impacts of GI programs could be maximized with multilevel supports, such as financial mentoring, peer support, and immediate service referral, to elevate emerging adults’ capacity to attain the financial stability required to support their mental and physical health (44–46).

To address these gaps in scientific knowledge, we designed a randomized controlled crossover trial to assess the efficacy of a multilevel GI intervention among low-income BEA to improve the immediate and future mental and physical health outcomes of BEA and to support BEAs’ agency and independence. Recognizing of the developmental needs of emerging adults, the intervention will provide access to financial capability programs through mentoring (individual level) and peer discussion groups (interpersonal level) in addition to providing BEA with GI. Furthermore, we lack critical knowledge of if and how the impacts of GI are sustained (47). To address this gap, this study will also evaluate outcomes one-year after GI payments end and explore potential mediators, contributing to knowledge of the impact of GI on BEAs’ trajectories over time. Finally, being that this is one of the first GI studies with emerging adults, we will closely track social harms to ensure that we can report on any potential downsides of providing GI to emerging adults. Notably, there is more speculation around potential negative effects, such as de-incentivizing work, than empirical data to support these concerns.

Although efforts to address the health impacts of structural racism through public health interventions have grown (48), the literature on incorporating anti-racism praxis into research methodology has been limited (49), focusing on certain areas such as conceptualizing race or community engagement. Anti-racism praxis is the reflexive process of dismantling racism in actions and systems (50). Failure to critically consider race and equity in study design and implementation can inadvertently perpetuate structural racism through research. We prioritized racial equity in our approach; we share examples of how we sought to weave anti-racist approaches into the design of the GI intervention, the design of the evaluation, and implementation of the project.

## Methods

### Trial design

**Figure 1:**
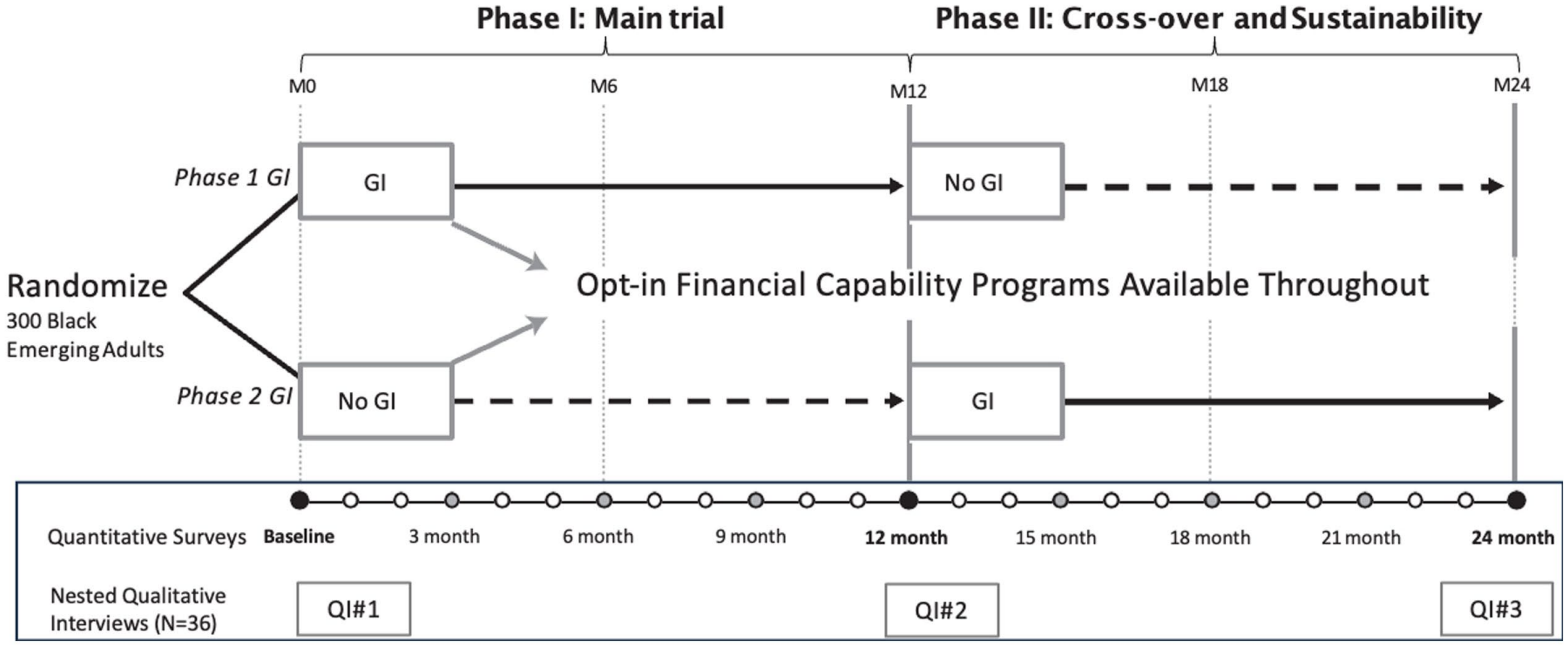
BEEM project study design.

The BEEM trial design is mixed methods, comprised of the quantitative randomized controlled crossover trial with a complementary, nested qualitative component including longitudinal semi-structured in-depth-interviews with a subset of participants to explain the processes that drive the results of the intervention (see Figure 1) (51). We aim to determine the impact of GI on three related sets of primary and secondary outcomes: (a) financial well-being (investing in education and training, savings, debt, financial capability); (b) mental health (depressive symptoms, anxiety, hope/future orientation); and (c) utilization of mental health and sexual and reproductive health (SRH) services. For the trial, 300 low-income BEA are randomized 1:1 to receive 12 months of GI either (a) during the first 12 months of follow-up (Phase I) or (b) in the second 12 months of a total of 24 months follow-up (Phase II). Most GI evaluations have used either parallel-group RCT designs, withholding GI from control groups, or rely on quasi-experimental (pre-post) designs. To address equity in the study, we employ a crossover randomized controlled design to ensure all participants in the project receive GI and offer peer discussion and financial mentoring to all participants. This allows us to implement a rigorous study design to draw causal inferences while centering BEA needs. The addition of opt-in financial capability programs allows us to explore whether complimentary supports can enhance GI while simultaneously respecting BEAs’ autonomy to choose when and how to engage with supports. An additional benefit of the crossover design is that it allows us to not only determine the impact of GI alone, but also sustained impacts once GI is concluded, thus addressing an important knowledge gap regarding sustainability in the evidence for GI programs. This protocol is written according to the Standard Protocol Items Recommendations for Interventional Trials (SPIRIT) 2013 statement (52), an international standard for trial protocols.

### Randomization and treatment allocation

Participants are randomized to either the “Phase 1 GI” or “Phase 2 GI” treatment arm by REDCap electronic data capture tools hosted at University of California, San Francisco. REDCap is a secure, web-based software platform designed to support data capture for research studies (53, 54). We loaded REDCap with a randomization sequence generated in SAS software 9.4 (55) using block randomization with varied permutations, with equal allocation to treatment arm and balanced by gender, age group, and city of residence. Due to the nature of the intervention, blinding the participants and study staff is not possible. Study staff inform participants of group assignment during the enrollment appointment, following baseline survey completion. The investigators are blinded to allocation, as is the trial methodologist (SBS), who will run the final analyzes.

### Eligibility and recruitment

Eligible participants must be between 18 and 24 years of age, identify as Black or African-American, have lived in the United States for at least 3 years, have no plans to leave the Bay Area permanently in the next year, not be currently enrolled in another GI project, and live in a Low-Income Housing Tax Credit (LIHTC) Qualified Census Tract (QCT) in San Francisco or Oakland, CA (tracts where 50% of households have incomes below 60% of the Area Median Gross Income (AMGI) or have a poverty rate of 25% of more) (56). Because BEA may live with parents and not know their household income, we utilize the census tract residential criteria as an indicator of low-income, which is standard for recent GI pilots in California. In addition, determination of income can be administratively burdensome for people; using census tracts as a proxy for income-level can reduce barriers for recipients. Given the structural inequities that drive housing access in Black communities, individuals who are unhoused and stay in San Francisco or Oakland are also eligible for participation, pending a letter from an agency serving unhoused young adults that can affirm their housing status.

Eligible participants are recruited from low-income areas in San Francisco and Oakland, CA, two cities with a history of residential segregation and racial economic inequities and which are grappling with gentrification and displacement of Black residents (57). Participants are recruited through partner agency outreach and direct strategies (e.g., flyers, word-of-mouth, street outreach). All interested individuals are directed to a web screening form on a public website;^1^ all those who apply and meet the age, race, and city criteria are asked to provide contact information. They are called or emailed to schedule a phone screening conducted by trained study personnel. After screening, eligible individuals are included in a participant pool. Participants are selected from the participant pool through a monthly, computer-administered drawing balanced by gender, city of residence, and age group (18–20 and 21–24).

### Intervention

**Figure 2:**
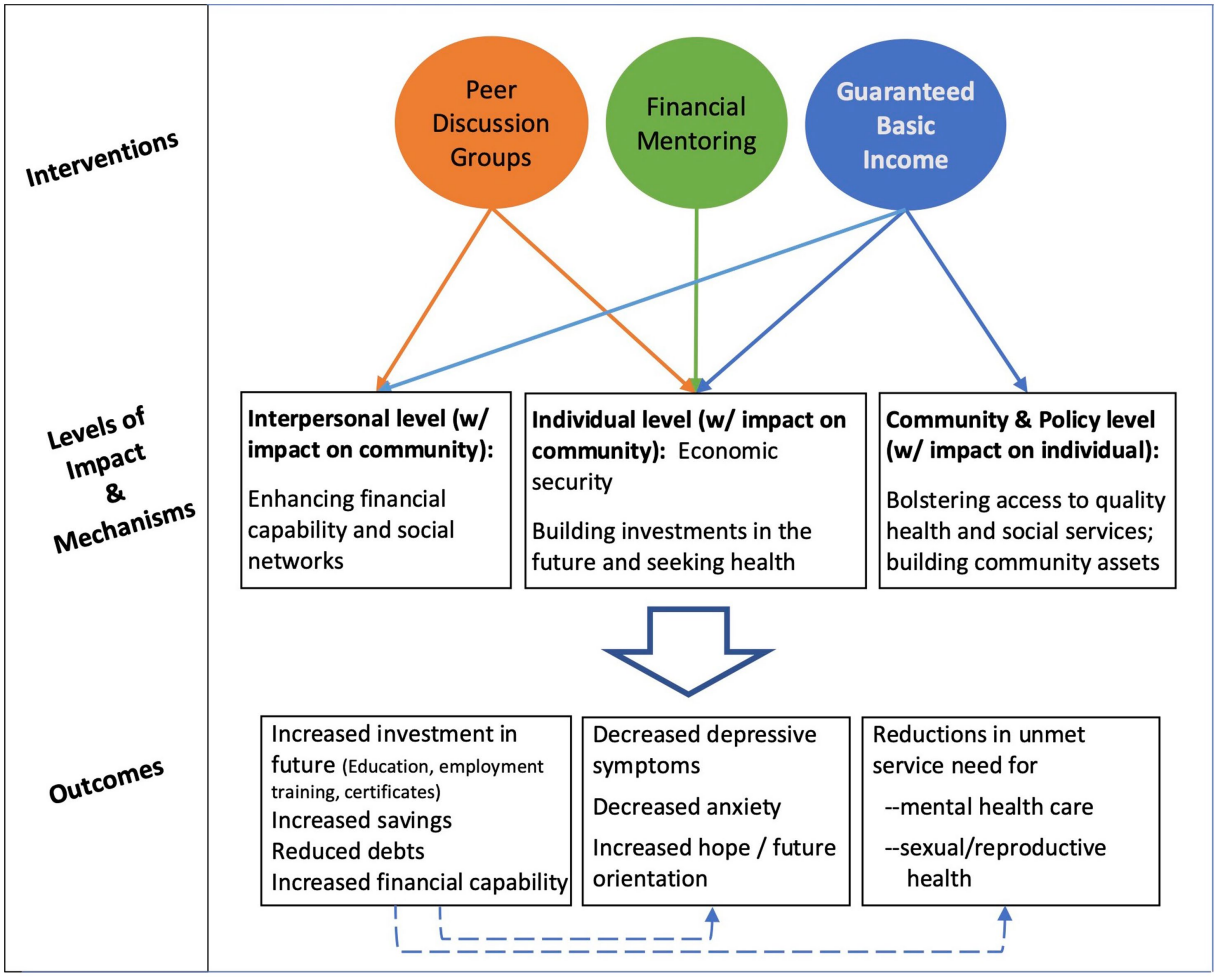
Conceptual model of the multi-level black economic equity movement intervention.

The intervention is carried out in partnership with MyPath, a national nonprofit whose mission is to seed and empower economic pathways for low-income youth and young adults, especially Black and Indigenous People of Color (BIPOC). The intervention includes three basic elements: guaranteed income, financial mentoring, and financial capability peer discussion groups–all of which are designed in consultation with community representatives (see Community Working Group described below). The intervention is designed to address multiple levels of influence on health by including components that address not only factors at the individual-level, but also those at the interpersonal-level and, when implemented at scale, community-level factors (see Figure 2). The key ingredient of this approach is that it enables BEA to create a personalized path to build wealth and health. Strategies to address health disparities have traditionally placed the onus on individuals to change. In contrast, GI – with no strings attached – aims to change a structural inequity and eliminates paternalistic indicators of ‘worthiness’ by valuing the autonomy and self-determination of all participants. In the same vein, participation in financial capabilities programming (i.e., financial mentoring and peer-support) in the project is optional and available to participants at any point during the project duration, ensuring that each participant has the right to define what is beneficial for them and when to engage (or not engage) in these options.

#### Guaranteed income (individual and community-level)

An income of $500 is distributed monthly over the course of 1 year, either in their first or second year of the two-year program. The GI distribution will be administered by Community Financial Resources (CFR) via a Focus Card. The Focus Card is a prepaid debit card with no monthly fees, no penalties, and no minimum balance, allowing participants to have immediate access to their funds (akin to a checking account) as well as the ability to save (akin to a savings account). Deposits are made on the first of the month.

#### Financial mentoring (individual-level)

The mentoring component is based on the model at MyPath, which incorporates a racial equity lens and addresses systemic barriers, such as a lack of quality financial products in under-resourced communities. Mentoring includes a blend of services that strengthen financial decision-making skills and access to quality financial products. Mentors are trained and experienced financial coaches with lived experiences that mirrors those of participants. Discussion topics are responsive to each participant and their priorities; however, mentors will ensure that participants understand the ‘basics’ before moving to more complex topics and will have access to a library of topics to support the young people in their varying needs. For example, a participant with a bank account and a budget will not need to review “budgeting and banking 101.” However, if they do not understand credit, the mentor will review this topic before supporting them to move forward with decisions affecting their credit. In past work with BEA, MyPath has identified priority areas, including building credit (credit repair, improving credit scores, credit products); savings (emergency savings plans, savings habits and strategies, understanding savings accounts); money management (creating budgets, understanding income vs. expense, assessing spending, making financial decisions that are aligned with personal goals and values); financial products (credit building products, auto loans, credit cards); and long-term goals (home ownership, investments). Opt-in mentoring sessions are co-scheduled by mentors and participants based on their availability and include up to six one-hour sessions conducted on-line.

#### Peer discussion and support groups (interpersonal-level)

Peer discussion groups, offered twice per month, emphasize financial capability skills and simultaneously create a space for participants to form bonds and seek support on these topics. Consistent with the literature indicating the strengths of peer support groups (58–61) and positive development (62, 63), the discussion groups aim to strengthen social networks and establish cohesion and support as peers reflect on their experiences, share strategies and resources, problem-solve, learn from each other, provide emotional support, hold each other accountable, and experience a sense of belonging. Groups of up to 10 participants are co-facilitated by a financial mentor and a near peer (Black young adult aged 22–27 with extensive financial capability program experience), meet for 1 h, and cover financial content.

### Community working group

All components of the intervention and much of the research design, including the data collection instruments, has been co-designed and is monitored by our Community Working Group (CWG). The CWG is comprised of partnering community agency representatives, city officials involved in health and wealth generation programming, the investigative team, and young adults or near peers. Community involvement was key in selecting a study design that ensured all participants would have access to guaranteed income (that is a wait-list design vs. a standard parallel arms trial). Similarly, the CWG collaborated in the design of the drawing for participant selection–ensuring equity among agency-affiliated and non-agency affiliated participants in that those with fewer connections were not disadvantaged by a first come, first serve model. The CWG also selected the project logo in conversation with the study team and graphic designer and weighed in on the website content and design. The CWG met monthly during project planning and start-up and will meet once every 2 months throughout the remainder of the project. They will also be integrally involved in developing and implementing a dissemination plan, identifying any community concerns with the research, and interpreting and framing the research findings and representation of BEA. CWG members are provided monetary compensation for their participation and time.

### Research hypotheses

Our primary research question is to understand the potential impacts of GI on BEAs investment in their future (education, employment, training), mental health (depressive symptoms), and unmet mental health (MH) and sexual/reproductive health (SRH) service needs. We are also interested in learning if complementary financial supports propel greater impact on health outcomes and understanding to what degree GI impacts dissipate after the transfers end. Thus, our hypotheses are as follows.

*H1*: *GI will improve all outcomes*. Youth receiving GI in phase I will have greater increases in investments in their future (H_1a_), greater decreases in depressive symptoms (H_1b_), and larger reductions in unmet need for MH and SRH services (H_1c_) at 12 months than those who do not received GI in phase I.

*H2*: *GI + financial supports will improve outcomes more than GI alone*. Participants receiving GI in phase I who participate in mentoring or peer discussion groups will have greater increases in investments in their future (H_1a_), greater decreases in depressive symptoms (H_1b_), and larger reductions in unmet need for MH and SRH services (H_1c_) at 12 months than those not opting into financial support programs.

*H3*: *Gains from GI will be sustained*. Participants receiving GI in phase I will not experience a decline in odds of enrollment in educational/training programs (H_3a_) or an increase in odds of depressive symptoms (H_3b_) or unmet need for MH and SRH services (H_3c_) following the removal of GI (between month 12 and 24).

*H4*: *Accessing complementary financial supports prior to receiving GI will improve overall outcomes.* Among BEA who do not receive GI in phase I, those who engage in mentoring or peer discussion groups in phase I will have greater increases in investments in their future (H_4a_), greater decreases in depressive symptoms (H_4b_), and larger reductions in unmet need for MH and SRH services (H_4c_) at 24 months than BEA who did not opt-in into the financial programming prior to receiving GI.

### Data collection and assessments

#### Structured surveys

We conduct comprehensive surveys annually and brief monitoring surveys of primary outcomes every 3 months, with some additional secondary outcomes and covariates collected at six-month intervals. Annual comprehensive surveys are conducted in-person at enrollment, 1 year (point of cross-over) and 2 years. The annual survey will assess primary and secondary outcomes as well as covariates. Brief 3-month surveys are completed online and focus on primary outcomes and any change in demographics, such as living situation. Links for the brief 3-month surveys are sent by SMS or email, depending on participant preference, up to four times within a 10-day window, with a final personalized reminder sent 1 day before the link expires. All surveys–both comprehensive and brief–are collected in REDCap by CAPI (Computer-assisted personal interviewing) in order to ensure privacy and consistency in data collection. Soft credit checks require an additional consent and are performed to assess participants’ baseline credit scores, following enrollment, and again at 6 and 12 months. Participants are remunerated for all surveys via their preferred form of payment (CashApp or gift card). Centering BEA needs and preferences, payment options were determined in consultation with BEA and the CWG.

#### Monthly check-ins

Participants receive monthly SMS prompts or emails (based on participant preference) that will query employment, earnings, and service needs for psychological counseling or SRH. The check-ins promote both engagement and retention of the cohort and provide an immediate, targeted, and relevant response or referral to services at or near when participants experience distress or needs. Like surveys, check-in links are sent up to four times. To support compliance, participants are provided $100 for responding to at least 80% of the monthly prompts sent. Finally, the team also sends monthly text messages to remind participants about financial capability program opportunities or to let participants know about upcoming events, such as job fairs.

#### Monitoring intervention uptake

Uptake of financial capability options are monitored via electronic reporting forms, entered into a Salesforce database, which is used to record attendance at peer discussion groups and mentoring sessions using participant ID, date, and activities completed. We monitor GI payments via the CFR card to ensure monthly payments were disbursed. We continually examine intervention activity and project milestones to assess observed vs. expected enrollments, uptake, and continuation of activities, with pre-specified timepoints for comparison and decision making. For example, should we find that fewer than 40% of enrolled participants are engaging in the peer discussion groups, this will trigger a review discussion with staff and the CWG to determine if additional recruitment or training efforts are needed, if there are structural barriers lowering attendance that need to be addressed (e.g., childcare needs, night school), and whether some programming should be re-oriented.

#### Qualitative data collection

We conduct three in-depth interviews over a period of 2 years (following enrollment, 12 months and 24 months) with 36 purposively selected participants, for a total of 108 interviews. Potential participants are identified by MyPath staff at enrollment and qualitative researchers select and contact potential participants to balance the sample by “Phase 1 GI” vs. “Phase 2 GI,” residential location (Oakland vs. San Francisco), gender, as well as other emergent criteria based on ongoing data collection (i.e., parenting status). The first interview is conducted within 2 months of enrollment; the second and third interviews will occur 1 and 2 years later, allowing us to capture the period before GI receipt, when GI ends, and a year after GI has ended. The first interview will focus on current living situation, education and employment status; personal and financial aspirations, including personal goals related to GI support; community involvement and support; family relationships; and current practices around physical and mental health, including access and utilization of health services. The second and third interviews will focus on changes in living situations, educational or employment opportunities, peer networks, personal and financial goal achievement, and experiences with GI and the multilevel supports. The third interview will also provide a critical understanding of experiences after GI has ended. Interviews will query about involvement in all financial capability programs, involvement in community building, and access to physical and mental health services. The 90-min interviews are conducted by trained qualitative researchers, digitally recorded and transcribed.

### Retention

Participants who do not respond to two sequential check-ins, or have been out-of-touch with the project for 3 months, will receive a personal phone call from project staff to strengthen participant engagement, check how they are doing, and refer to any needed services. Staff will attempt three calls, texts, or emails within a week prior to reaching out to alternate participant contacts, including family, friends, or agency contacts whose name the participant provided at the enrollment visit. Alternate contacts are asked for updated contact information if the participant’s number has changed and to let the participant know we are trying to reach them. We also attempt to contact the participant through social media if the participant provided their username. If no contact is made, we wait 1 month and try again, including alternate contacts. We track all communications, including SMS sent and received, and retention calls, through our REDCap software platform. Because mobility may be an issue in this population, non-response will not be considered tacit refusal in perpetuity. Non-responsive participants will only be considered “lost to follow-up” at the conclusion of the study (or if they request to be removed from study participation). Participants provide multiple forms of contact info (phone, social media, family/friend contact info, agency contacts) to facilitate communication and connection with the project.

### Ethics

This study, consent forms, and all study procedures have been approved by the University of California, San Francisco Institutional Review Board (IRB #:21–35420). All participants undergo consent to participate in the study, including provision of personal contact information. Participants’ personal information is recorded and stored privately on a secure, password-protected online server. Investigators, project staff, and authorized personnel (i.e., representatives of the sponsoring agency, implementing academic institutions) may review data for monitoring the project. De-identified data may be shared with other researchers for future studies upon reasonable request; personal or potentially identifying information will not be shared. The principal investigators have no competing interests. Any protocol amendments, including outcomes or analyzes, are subject to IRB approval and will be updated in the U.S. National Library of Medicine clinical study registry (ClinicalTrials.gov). Participants will be informed of protocol amendments if terms of participation are affected.

Safety oversight is under the direction of a Data and Safety Monitoring Board (DSMB) composed of three independent investigators with appropriate expertise, including public health, epidemiology, adolescent psychology, cash transfer programming, and health economics. The DSMB met prior to study start, at 6 months into recruitment, will meet again after 1 year of programming, and at least annually thereafter to assess safety data. The DSMB operates under the rules of an approved charter. All serious or unexpected adverse events that are possibly related to study participation will be assessed by the principal investigators immediately upon report and are reported to the UCSF IRB and DSMB within 5 working days of UCSF PI awareness. The DSMB may suggest that the study be modified but will not stop the study. Discontinuing the study would deny enrolled participants the full amount of anticipated GI (for the waitlist group), which is unethical and could inflict harm on participants.

Standard operating procedures (SOP) for distress have been developed and study staff have been trained to address participants’ reports of suicidality, mental distress, or abuse. On surveys and check-ins, reports of suicidality, mental distress, or unsafe relationships trigger an automated alert to the study staff. Participants may also directly report these events to study staff in qualitative interviews, mentoring, or peer discussion groups. Staff contact participants when alerts are received to assess the situation and precede according to protocol. Depending on the participant’s stability and request, staff may contact an on-call clinician to speak to the participant, may refer the participant to services, or may agree to a follow-up call at a later date. All events and staff responses will be recorded for study monitoring.

### Measures

#### Exposure

The primary exposure is intervention arm assignment (see all measure in Table 1). Secondary exposure variables include: receipt of financial mentoring (yes/no) and participation in peer discussion groups (yes/no) during each 3-month study interval. Because engagement with any treatment option will vary by individual, we will also capture intervention dose (e.g., mentoring sessions attended) on study monitoring forms.

**Table 1:** Study measures: domains, instruments, and time points.

Domain	Instrument/measure	Data source and frequency
Primary exposure		
Intervention	Randomization arm (GI receipt in phase I or phase II)	Study records
Primary outcomes		
Financial well-being	Investments in future (Enrollment in education/training)	Quarterly surveys
Mental Health	CESDR-10: major or probable major depressive episode (64, 65)	Quarterly surveys
Unmet need for health services	Unmet need for mental health services	Quarterly surveys and monthly brief check-ins
	Unmet need for sexual and reproductive health services	Quarterly surveys and monthly brief check-ins
Secondary outcomes		
Financial well-being	Savings, Debt	Surveys at baseline, 6, 12, 18, 24 months
	Credit score	Assessed at baseline, 6, and 12 months
	Financial Capability (knowledge, skills, practices, mindset) (45)	Every six months: baseline, 6, 12, 18, 24 months
Mental health	Anxiety – moderate–severe: score ≥ 10 on GAD-7 (66, 67)	Quarterly surveys
	Hope/ future orientation (68, 69)	Surveys at baseline, 6, 12, 18, 24 months
Physical health	General health (70); fatigue	Surveys at baseline, 6, 12, 18, 24 months
Secondary exposure		
Intervention dose	Engagement with optional intervention components (peer groups and mentoring)	Study monitoring forms
Covariates/Mediators		
Demographics	Age, gender identity, housing/living situation, household membership, income, employment	Quarterly surveys
Strengths and assets	Resilience (71); self-esteem; Black identity (72); decision-making and goal-setting	Frequency varies – six-month and annual surveys
Social and structural contextual factors	Experienced discrimination (73); alcohol and substance use (74); social support (75, 76); social cohesion (77, 78); incarceration; relationship violence (79); trauma (80); prosocial involvement; food security	Frequency varies - quarterly; six-months; and annual surveys

#### Primary outcomes

Financial well-being: We measure investments in the future, specifically by measuring participants’ enrollment in formal education, leadership, or employment training programs during each 3-month study interval. This indicator of wealth generating investments in the future has been utilized in United States studies (34) and represents potential long-term gains, despite the intervention being time-limited. We also collect data on the names of programs, time spent on these activities, the duration enrolled, as well as engagement in informal or self-directed learning. This outcome is an intentional acknowledgement of the agency, assets and resilience of BEA, rather than simply focusing on deficits or vulnerabilities.

#### Mental health

We assess major or probable major depressive episode in the past week at each 3-month study interval using the Center for Epidemiologic Studies Depression Scale Revised (CESDR-10). This measure uses an algorithm that accounts for both symptom severity and duration to determine occurrence or probable occurrence of a major depressive episode and has been validated in young people (64, 65).

#### Unmet health service needs

Defined as the absence of care seeking when needed, unmet mental health (MH) need will be assessed by indication of major or probable major depressive episode on the CESDR-10 or moderate or severe anxiety (score of ≥10) measured by the Generalized Anxiety Disorder 7-item scale (GAD-7) (66, 67) and not reporting that care was utilized in the past 30 days. Service utilization is based on self-report as stating “no” to having accessed any MH service from any kind of health professional (e.g., mental health specialist, general practitioner, nurse, psychiatrist, psychologist, counselor, or social worker) in either an individual or group therapy format. We will assess unmet need for SRH services, which will be measured as self-reported need for SRH (having symptoms of a sexually transmitted infection, unprotected sex, or being at risk of unwanted pregnancy) and not reporting that SRH services were utilized in the past 30 days.

#### Secondary outcomes

Secondary financial outcomes include savings (having enough to cover an unexpected expense of $400) (81); holding of debt (including fraudulent debt, owed child support, banking/overdraft fees, bail debts, school debts, utility debts, and credit card or payday loan debt); credit score; and financial capability, a composite measure of financial knowledge, skills, and practices (45) that is sensitive to rapid change following similar financial capability interventions (82, 83). In addition to self-reported savings and spending, we will utilize aggregated data from the CFR Focus Card to assess spending, balances, and savings by treatment group as well as by engagement in financial capability programming. We will also examine intensity of the measures that comprise the primary financial well-being outcome (time invested in education, certification, and employment training program participation).

Secondary mental health outcomes include moderate or severe anxiety indicated by a score ≥ 10 on the GAD-7 (66, 67) and hope/future orientation measured by the Hope Matters Scale (68, 69). Secondary physical health outcomes include general self-rated health (70) and fatigue (84).

Covariates include: socio-demographic characteristics, including education, employment, income, partnerships, household membership, living/housing situation, and financial supports. We will also assess BEAs’ strengths and assets, and other individual, social and structural contextual factors that could act as potential confounders, could modify the effect of the intervention, or could mediate the intervention’s impact on mental and physical health.

### Analyses

#### Preliminary analyses

Frequency tables for all categorical variables and measures of central tendency and variability for continuous variables (e.g., average monthly income, age) will be used to characterize the sample and check for imbalances between the randomization arms. If arms differ significantly at baseline on one or more covariates or if imbalances in arms occur due to differential loss to follow-up, we will use causal modeling methods (e.g., inverse probability weighting, targeted maximum likelihood estimation) to balance distributions and obtain the desired marginal effect estimates under the counterfactual assumption of balanced arms (85–89). We will address incomplete data with multiple imputation (90). SAS will be used for analyses.

#### Primary analyses

Our primary analyses will test the hypothesis that GI will improve all primary outcomes at 12 months (H1). Specifically, we hypothesize that participants receiving GI in phase I will have (a) greater increases in investments in their future; (b) greater decreases in depressive symptoms, and (c) larger reductions in unmet need for MH and SRH services at 12 months than the no-GI arm.

We will use generalized estimating equations (GEE) to test our primary hypotheses and this analysis will follow an intent-to-treat approach by including all randomized participants. Our primary interest is to estimate the marginal or population-average effects of intervention participation on these primary outcomes rather than the effect for a hypothetical average subject (91). Moreover, within-subject outcome correlations are considered nuisance parameters rather than quantities of interest to be modeled explicitly. Accordingly, GEE can be used to estimate the marginal effects using difference-in-differences (DID) comparisons of post-baseline (follow-up) measurements of the Phase 1 GI group with the comparison arm in the main trial Phase. Since our primary outcomes are dichotomous, these models will employ a logit link and assume a binomial distribution. For each model, we will include indicators for study arm, time and the interaction between study arm and time to examine the relative change in outcomes for each arm during Phase 1. If changes in each outcome do not follow a linear trend, we will explore alternative relationships including polynomial and spline terms. Alpha (α) will be set at 0.05 for each of our planned comparisons. Any additional post-hoc comparisons (e.g., paired comparisons of groups at each time point) will maintain a nominal alpha of 0.05 through the use of simulation-based stepdown multiple comparison methods (92).

Though GEE estimates are consistent even if the correlation structure is mis-specified, GEE’s statistical efficiency improves as the working correlation structure more closely approximates the actual correlation structure (93, 94): therefore, various correlation structures suitable for the study’s design will be considered (e.g., exchangeable; M-dependent) (94). The QIC statistic will be used to select the final correlation structure (95). Additional covariates, including baseline demographic characteristics, psycho-social covariates and social and structural contextual factors will be included if they improve QIC. Robust Huber-White “sandwich” standard errors will be used to obtain correct inferences even if the chosen correlation structure remains slightly misspecified. GEE case deletion diagnostics (e.g., DFBetas, Cook’s D) will be used to investigate whether influential cases are present; if so, results will be reported with and without influential cases included (96).

#### Sample size estimation

Power analyzes were generated using the equation defined by Diggle et al. (97) to compute minimum detectable effect sizes for the primary analyses to address Hypotheses 1a-1c. The study will begin with 300 participants equally allocated to two study groups. Assuming 25% attrition over 12 months, we anticipate an average of 4.3 observations from 300 participants will be available for analysis of investments in the future and mental health outcomes and an average of 5.7 observations for analysis of unmet service need outcomes. Assuming *α* = 0.05 and power = 0.80, with four highly correlated post-baseline measurements [intraclass correlation (ICC) = 0.20–0.80] and a range of outcomes for waitlist control participants (change in each outcome between 5 and 40%), we will have sufficient power to detect differences as low as 5.7–10.7% for increase in enrollment in educational or training programs and decrease in depression, and 4.5–10.6% for decrease in unmet service need, respectively, in the intervention arm compared to waitlist controls.

#### Secondary analyses

We will determine if BEA receiving GI in Phase 1 who opt-in to peer discussion groups and financial mentoring will have larger increases in odds of investment in the future, larger declines in odds of depressive symptoms and larger declines in odds of unmet mental and sexual health service needs at 12 months compared to those who did not opt into these support programs (H2). We will use the same modeling approach as described above for H1 used to test H2 except that analysis will be restricted to a single arm of the study (those receiving GI in phase I or those receiving GI in phase II).

As in our preliminary analyses, we will employ causal inference methods to adjust for any differences between sub-strata [e.g., individuals who do or do not opt in to financial mentoring or peer discussion groups at baseline or due to differential loss to follow-up]. As in our primary analyses, we will use logistic GEE to model our dichotomous outcomes, and continuous GEE specifications for continuous measures. We will test H2 via DID comparison of post-baseline measurements in the Phase 1 GI group during Phase 1 (as in H1). All comparisons for secondary analyses (H2 through H4) will be tested at *α* = 0.05 per comparison.

We will test (H3)–BEA receiving GI in Phase 1 will not experience a significant decline in odds of enrollment in educational or training programs, a significant increase in odds of depression or a significant increase in odds of unmet mental and sexual health service needs following removal of the GI (between month 12 and 24)–using an interrupted time series (ITS) approach. For these ITS analyses, we will include indicators for phase, time and the interaction between phase and time to examine the slope in each outcome during Phase 1, the presence of an immediate change in each outcome after removal of GI (level effect), as well the change in slope for each outcome during Phase 2 (slope). If changes in outcomes do not follow a linear trend, we will explore alternative relationships including polynomial and spline terms.

Following completion of Phase 2, we will assess (H4)–whether participants who received GI in Phase 2 who enrolled in peer discussion groups and/or financial mentoring during Phase 1 will experience a larger increase in odds of investments in the future, larger declines in odds of depressive symptoms, and larger declines in odds of unmet MH and SRH service needs compared to participants who received GI in Phase 2 and did not enroll in the opt-in financial programming during Phase 1. Analyses for H4 will follow the same analytical approach as described in H2 except that this analysis will be restricted to participants receiving GI in Phase 2. We will test H4 via DID comparison of post-baseline measurements in the Phase 2 GI group during Phase 2 (as in H1).

#### Exploratory mediation and moderation analyses

We will conduct a number of exploratory mediation and moderation analyses. For example, in mediation analyses we will investigate whether covariates, such as food insecurity, or interim financial well-being outcomes, such as debt, credit, or income, measured at 3, 6, and 9 months mediate the relationship between intervention assignment and subsequent mental health outcomes (i.e., less anxiety and depressive symptoms) measured at 6, 9, and 12 months. We will also investigate whether changes in financial and mental health outcomes mediate the relationship between intervention assignment (GI) and reduced unmet service needs. Finally, we will explore whether intervention impacts are different by gender, parenting status, or living situation (moderation).

### Qualitative data analysis

Using thematic analysis to guide the process, the team will develop a codebook based on deductive themes (98), based on topics captured within the interview guides such as current living situation, financial aspirations, community involvement, and goals related to GI support and inductive themes, which emerge within the data. A preliminary codebook will be developed, and then the team, consisting of the interviewers, MyPath staff, and a senior investigator, will select 3–5 transcripts to code together, refining the codebook through discussion, and developing definitions and rules around code application. Coder agreement will be assessed by having each member code a portion of a transcript, comparing the total number of segments coded to the number of coded segments where analysts agreed on code application to ensure that the analysts achieve a coder agreement threshold of 90 percent. Analysis team members will discuss any discrepancies in coding approaches to resolve differences and will repeat the process to build coder agreement until the threshold is met. Once the threshold has been achieved, the transcripts will be entered into Dedoose, an online qualitative data analysis program, and the remainder of the data will be coded. This process will be repeated with every wave of data collection, as the codebook is further refined with each iteration of the interviews.

### Dissemination

Overall findings will be disseminated to the local community, including study participants and their families and communities, via dissemination meetings. Dissemination will occur first following baseline data collection, including descriptive analysis to understand the context and circumstances of our participants, next after the end of Phase 1 (H1 and H2) to report primary trial outcomes, and again following Phase 2 (H3 and H4) as results from the secondary trial outcomes become available. Dissemination to the scientific, programmatic, and policy research communities will include presentations at scientific meetings, publications in peer-reviewed journals, and fact sheets for policymakers.

### Anti-racist approaches to implementation

Historical exploitation of Black communities in medical research requires centering anti-racism in public health research, including challenging institutional norms and policies. We utilized many additional strategies to prioritize equity and center the needs of BEA through the study’s implementation. To maximize investments in the participants themselves and ensure that academic institutions do not unfairly profit from work to benefit communities of color, the BEEM project requested a waiver of institutional overhead costs associated with GI payments, which we successfully obtained from the university. Other anti-racist efforts employed by the project also required institutional change, such as advocating for and pursuing options to provide participant incentives for survey completion in the form they preferred (e.g., CashApp), which was not initially endorsed by institutional policy, but was permitted as a pilot program. The project has also maintained a very active CWG and compensates the members of our CWG for their valuable time and input. We also seek to minimize harms to participants by preventing loss of social service benefits and loss of GI payment in the form of taxed income. We consulted legal experts to successfully establish the legal basis for classifying GI as a gift and not taxable income. We have also secured state waivers to protect benefits such as CalFresh and CalWorks and included free provision of benefits counseling to all participants to address concerns about losing other benefits due to receipt of GI.

## Discussion

The BEEM Project addresses the important impact of decades of structural racism and inequitable access to opportunity and wealth generation for BEA, while also addressing scientific knowledge gaps regarding the impacts of GI on financial well-being and health outcomes. The randomized controlled crossover design supports the ability to draw causal inferences while ensuring all participants have access to no-strings-attached cash payments. This study will illuminate the impacts of complementary financial capability programming (i.e., mentoring, peer discussion groups) and their ability to bolster the effects of GI as an accompanying policy program. The extended data collection period will aid understanding of sustained impacts of GI one-year after payments end, filling a critical gap in knowledge on the degree to which the impacts of GI dissipate (follow-up for most GI projects have rarely extended beyond a few months). Results about the extended impact of GI can also provide evidence about augmenting health trajectories over the life course.

Importantly, results of the study will also help determine the utility of GI as a strategy to tackle racial health disparities. The focus of our project is on empowering low-income BEA, a group that has been made vulnerable by a history of intersecting social, economic, and political systems that result in lasting economic inequities and denied opportunities. This study will provide evidence about targeting GI within a group whose financial vulnerability and health inequalities have been created by structural racism and impact health transitions to adulthood. We apply anti-racism praxis and actively engage with communities, an approach vital for informing our approaches to affirming the autonomy of BEA, allocating and distributing resources, and challenging institutional (including research) norms.

This study has some limitations. Little is known about the unintended consequences of GI among emerging adults, but we are gathering information, such as social harms, to shed light on potential limitations of GI. The BEEM Project uses residence in low-income census tracts (or marginal housing status) as a proxy for low-income status since many young people do not know their household’s income. This may result in greater heterogeneity in baseline income levels than directly measurement of household income. However, we will assess other indicators of income and socioeconomic status (receipt of public benefits, maternal education, income earned) and can adjust for baseline differences if treatment arms are imbalanced. Selection bias may be introduced due to self-selection into the non-GI intervention components (financial mentoring and peer discussion groups). We will adjust for these differences across sub-strata in our analysis by using causal inference methods. The ITS analysis of H3 will be subject to the usual threats if other time shocks occur, such as changes in local social determinants of health outside of study control; these conditions will be monitored to assist in results interpretation. Because this study is set in two cities in California, findings may not be generalizable outside of urban areas in California. Finally, the temporary one-year nature of our GI intervention may result in smaller effects than would an ongoing GI program supporting BEA throughout their entire emerging adulthood period from age 18 to 24.

Acknowledging that structural racism drives racial health disparities, this study focuses on GI as a strategy to address the *effects* of structural racism on individuals’ health outcomes, rather than intervening on structures or institutions that perpetuate racial inequities. More investigation is needed to understand how GI may influence individuals’ interactions with structural factors. For example, it is plausible that this intervention could impact communities or policy change, especially since GI receipt has been associated with greater perceived ability to influence social change (99). Future research could examine GI recipients’ involvement with broader structural change or investigate spillover effects at neighborhood, institutional, or policy levels. On the other hand, given that structural racism has also shaped neighborhood environments via systematic disinvestment, it is also plausible that contextual neighborhood effects may potentially augment the impact of GI via resources or social capital. Future research should assess heterogeneity of treatment effects to discern whether certain contextual factors can be targeted for intervention.

## Conclusion

Amidst growing interest in deploying GI as an approach to increase economic security, the BEEM Project is one of the first GI trials that will be rigorously evaluated for health outcomes among low-income Black emerging adults during their transition to adulthood and independence. By sharing the replicable approaches and strategies employed by the BEEM project, we hope to provide insight and examples for researchers committed to addressing health disparities and structural racism.

## Author contributions

SL: Conceptualization, Funding acquisition, Investigation, Methodology, Project administration, Supervision, Writing – original draft, Writing – review & editing. MKL: Conceptualization, Funding acquisition, Investigation, Project administration, Resources, Supervision, Writing – original draft, Writing – review & editing. MN: Writing – original draft, Writing – review & editing. AA: Project administration, Supervision, Writing – review & editing. MB: Project administration, Writing – review & editing. ARM: Formal analysis, Methodology, Writing – review & editing. EA: Formal analysis, Investigation, Methodology, Writing – review & editing. SS: Investigation, Methodology, Writing – review & editing. FQ: Conceptualization, Writing – review & editing. AD: Project administration, Writing – review & editing. AM: Project administration, Writing – review & editing. WD: Conceptualization, Investigation, Writing – review & editing. MAL: Conceptualization, Funding acquisition, Investigation, Supervision, Writing – original draft, Writing – review & editing.

## Footnotes

^1^beemproject.org

## Abbreviations

AMGI: Area Median Gross Income
BEA: Black Emerging Adults
BEEM: Black Economic Equity Movement
BIPOC: Black and Indigenous People of Color
CFR: Community Financial Resources
CWG: Community Working Group
DID: Difference-in-differences
DSMB: Data Safety and Monitoring Board
GEE: Generalized Estimating Equations
GI: Guaranteed Income
IRB: Institutional Review Board
ITS: Interrupted Time Series
LIHTC: Low-income Housing Tax Credit
MH: Mental Health
SRH: Sexual and Reproductive Health
QCT: Qualified Census Tract
